# Investigating sex differences and age of onset in emotion regulation, executive functioning, and cannabis use in adolescents and young adults

**DOI:** 10.1186/s42238-024-00225-z

**Published:** 2024-04-26

**Authors:** Natasha E. Wade, Kelly E. Courtney, Alexander L. Wallace, Laura Hatz, Joanna Jacobus

**Affiliations:** Department of Psychiatry, University of California, San Diego, 9500 Gilman Drive, MC 0405, La Jolla, CA 92093 USA

**Keywords:** Cannabis, Emotional functioning, Executive functioning, Age of onset, Sex

## Abstract

**Background:**

Young adults have historically high levels of cannabis use at a time which coincides with emotional and cognitive development. Age of regular onset of cannabis use and sex at birth are hypothesized to influence the relationship between cannabis use and cognition. Here we investigated past 6-month cannabis use in relation to emotional and executive functioning. We further considered age of onset and sex in subgroup analyses.

**Method:**

Young adults (*N* = 225; ages 16–22) completed a substance use interview and cognitive battery, including the Emotional Word-Emotional Face Stroop and NIH toolbox executive functioning tasks. Linear regressions examined relationships between past 6-month cannabis use episodes and performance. Subgroup analyses investigated whether age of onset or sex impacted relationships.

**Results:**

After correcting for multiple comparisons, greater past 6-month cannabis use episodes were related to poorer Emotional Stroop Congruent Accuracy (*p* = .0004, FDR-*p* = .002) and List Sorting Working Memory (*p* = .02, FDR-*p* = .10) performance. Younger age of regular use onset marginally related to lower Emotional Stroop Congruent Accuracy performance (*p* = .03, FDR-*p* = .13). There were no cannabis use by sex interactions on cognition.

**Conclusions:**

Consistent with prior findings, results suggest small reductions in cannabis-related performance in processing speed during emotional Stroop and working memory tasks. Age of onset was modestly related to Stroop performance, but not sex. Longitudinal studies which detail patterns of cannabis and other substance use are needed to better assess brain-behavior relationships and other factors (e.g., age of onset of regular use, sex) which could influence cannabis-related impairments in cognitive functioning.

## Introduction

Adolescent and young adult cannabis use is associated with frontolimbic brain circuit disruptions as evidenced by decreased neural connectivity and reduced white matter tissue integrity in individuals with regular cannabis use [1–4]. These regions underly reward pathways and are crucial for effective neurocognitive functioning, including emotional control and executive functioning [5]. Yet the association between cannabis and these higher order cognitive functions is not always consistent [6–8]. Given that cannabis use reached peak prevalence rates among 18-30-year-olds in 2021 [9], understanding how cannabis is associated with cognitive outcomes and subgroups (e.g., by sex, age of onset) that may influence cannabis-cognition relationships in emerging adults is important, particularly in an evolving and growing cannabis product market.

Neurodevelopment, including structural and functional brain maturation, persists through the third decade of life [10, 11]. These changes include connections between prefrontal and limbic (i.e., frontolimbic) regions, which are key for emotional development and reward system processing [12]. Cannabis use during this time has been linked frontolimbic disruptions [1, 3, 13] and, more broadly, to a range of executive dysfunction [14, 15] and emotional control deficits [13, 16, 17], though a recent review of this topic area suggests smaller-scale reductions in cognition in the absence of persistent and heavy cannabis use during adolescence [18]. Notably, the majority of work in this area has utilized neuroimaging to indirectly assess emotional control, therefore it is important to assess the downstream behavioral impact of cannabis use in this domain as well as prefrontal functioning more broadly through executive functioning.

Several behavioral and biological factors are postulated to relate to decrements in cognition in those with cannabis use, which may partially explain prior inconsistent findings. Age of regular cannabis use onset (e.g., weekly use) has been linked to executive dysfunction [19] and poorer planning abilities [20]. Females are purported to have a particular vulnerability to cannabis use [21] during neurodevelopment that is related to worse performance on attention and executive functioning tasks [16] and altered functional connectivity during affective processing [13], amongst other cognitive domains like memory [22]. This may be due to females exhibiting greater acute cannabis-use effects in both high and cannabinoid concentration [23]. While female sex and young age of initiation are routinely thought to convey heightened risk to the deleterious effects of cannabis use on neurodevelopment, these risk factors are not consistently studied in emerging adult cannabis studies.

Given the evidence for frontolimbic disruption in those who use cannabis, here we aim to investigate emotional control using a newly validated Emotional Stroop task [24] and executive functioning tasks from the NIH Toolbox in adolescent and young adults who use cannabis. Regression analyses will examine whether past 6-months cannabis use relates to cognitive performance across tasks. We further include consideration of other important potential factors in cognitive performance and cannabis use: namely, we group by age of onset of regular cannabis use and provide comparisons by sex.

## Methods

### Participants

Participants from an ongoing study in San Diego, California, were included in the present analyses. They were recruited via flyers posted around local colleges and universities and through online advertising. Interested participants called a laboratory phone for verbal consenting and screening to assess eligibility. Eligible participants included 129 participants ages 16–22 who endorsed current, regular (at least weekly) cannabis use and 96 participants of the same age who did not use cannabis regularly (< weekly) in the past six months. Nicotine and tobacco product (NTP) and alcohol use were assessed across both those who use cannabis and controls.

### Exclusion criteria

Participants were excluded if they reported: excessive prenatal alcohol (maternal use of > 2 drinks per occasion, > 4 drinks in a week), tobacco, or drug exposure; premature birth (< 34 weeks gestation); other gestational or perinatal complications, including low birth weight (< 5 lbs); history of serious medical or neurological problems; head trauma with loss of consciousness > 2 min; current or past DSM-5 diagnoses other than cannabis or nicotine use disorder; learning disability; current use of psychotropic medications; non-correctable vision/hearing difficulties; not fluent in English; pregnancy; use of alcohol or cannabis within 12 h of study visit which would indicate potential current intoxication [25–27].

### Procedures

Eligible participants attended a 4-hour in-person study session which included cognitive testing, substance use interviews, and magnetic resonance imaging (data presented elsewhere [28, 29]). Participants were abstinent from all drugs other than nicotine on the day of the appointment (i.e., 12 h of required abstinence), with abstinence confirmed via toxicological testing. Participants gave written informed consent accordance with the University of California, San Diego Human Research Protections Program.

### Measures

#### Neurocognition

##### Emotional stroop

Participants completed the Emotional Word-Emotional Face Stroop (EWEFS) [24], in which participants were presented with emotional words overlaid on emotional faces that were either congruent or incongruent. They then sorted stimuli (emotional words) as either “good” or “bad”, while ignoring the image presented in the background. Four outcomes were measured: Congruent Accuracy, the proportion of congruent blocks that were accurately categorized; Incongruent Accuracy, the proportion of incongruent blocks that were accurately categorized; Congruent Response Time, the mean time (in milliseconds) to respond to congruent trials; Incongruent Response Time, the mean time (in milliseconds) to respond to incongruent trials. *NIH Toolbox.* For tests of executive functioning, the National Institutes of Health (NIH) Toolbox Cognition Battery [30] was used. Participants completed tasks on 3rd generation iPad Air devices (10.5in) under the administration of a trained research assistant. For the present study, included tasks measured domains of executive functioning: for set-shifting, participants completed the Dimensional Change Card Sort (DCCS) Test, a card sorting task wherein participants had to respond to changing rules; for inhibitory control and attention, they completed the Flanker Inhibitory Control and Attention Test, wherein participants responded to a displayed central target in the midst of a row of stimuli; for working memory, they completed the List Sort Working Memory Test, where they sorted audible and visual stimuli from smallest to largest; for episodic memory, participants completed the Picture Sequence Memory Test, wherein they recalled the sequence of visually displayed images; for processing speed, they completed the Pattern Comparison Process Speed Test, wherein participants quickly identified whether or not two images were identical. Uncorrected scores were used in the present analyses.

### Substance use history

Research assistants administered a modified version of the original Customary Drinking and Drug Use Record (CDDR) [31–34] to participants to collect detailed past year and lifetime substance use history. Substance “use episodes” constituted the primary variables of interest. Multiple use episodes of substances were logged if a participant had used more than once per day (e.g., in the morning, class in the afternoon, and before bed would equate to three use episodes). Additionally, other substance use variables of interest including self-report of age of onset of regular cannabis use and average cannabis use potency (for flower, reported as Low (< 5% THC), Medium, (10%), High (15%), Very High (20%+), or Don’t Know; for concentrate, Low (around 20% THC), Medium (40%), High (60%), Very high (80%+), or Don’t Know) were also collected. Age of onset of regular use was defined as the age at which a participant regularly (> weekly) used cannabis for at least a year.

### Toxicological assessment

Urine, oral fluid, and breathalyzer samples for alcohol were collected and tested to confirm self-report and abstinence from cannabis, alcohol, and other drugs. However, participants were allowed to use NTP as needed to prevent withdrawal effects.

### Sociodemographics

Participants self-reported sociodemographic characteristics (sex, race, ethnicity, education, maternal education as an estimate of socioeconomic status). While all were considered for inclusion in analyses, only sociodemographic factors that differed by past-month cannabis use status were included as covariates (i.e., sex at birth and race; see Sociodemographics in Results below).

### Statistical analyses

#### Groups and subgroups

Cannabis use groups were created and used only to identify covariates; primary analyses were run with cannabis use as a continuous variable. The cannabis use group was defined as those who regularly used (> weekly, or > 24 use episodes) cannabis over the past 6 months (range of use: 28 − 3,557 episodes); controls were those who did not regularly use cannabis (< weekly) in the past 6 months (range of use: 0–22 episodes). Additionally, subgroups were created and used in secondary analyses to assess the influence of age of onset of regular use or sex in relation to cannabis-cognition relationships. Age of Onset was defined as the first age at which an individual engaged in regular (> weekly for at least a year) cannabis use [35]. Age of onset subgroups consisted of those who had never regularly used cannabis (*n* = 98), those who regularly used at or after age 18 (*n* = 76), and those who regularly used before age 18 (*n* = 51), as 18 has previously been used as a cut-off for age of regular onset [35]. Participants were also separately assessed by sex at birth in order to determine whether cognition-cannabis relationships may be sex-dependent.

#### Primary analyses

All analyses were run in R version 4.1.0 [36] via RStudio [37]. Linear regression models using the “stats” package investigated past 6-months cannabis use (continuous use variable) in relation to emotional and executive functioning. Summary scores for each cognitive task were run in nine separate regressions: Emotional Stroop Congruent Accuracy, Emotional Stroop Congruent Response Time, Emotional Stroop Incongruent Accuracy, Emotional Stroop Incongruent Response Time, Card Sort, Flanker, List Sorting, Picture Sequencing, and Pattern Comparison. Covariates. Sociodemographic factors that differed by cannabis use group status were included as covariates (i.e., race, sex) as well as age, given the age range of participants and definition of age of onset of regular use. All analyses were run with cannabis use as a continuous variable by regression with past 6-month cannabis use predicting cognitive performance; no groups were used in the primary analyses. In addition to race and sex, past 6-month nicotine use episodes and past 6-month alcohol use episodes were combined and included as a continuous covariate representing history of other substance use. Multiple comparisons were adjusted within models using the “sjstats” package for false-discovery rate corrections. Data were checked for outliers with Cook’s d, with no outliers revealed. Statistical decisions were made if corrected p (FDR-*p*) < 0.05.

### Secondary analysis

Age of onset of regular cannabis use and sex at birth were separately considered in independent subgroup regressions. Subgroups were defined as listed in the Groups and Subgroups section above. Linear regression analyses were run assessing onset subgroup [no onset (never regular user) v. early onset (regular use before age 18) v. late onset (regular use at or after 18 years-old)] in relation to cognition, controlling for past 6-months combined nicotine and alcohol use, race, sex, and age. For analyses on sex-based effects, interaction terms between past 6-month cannabis use and sex were run in linear regressions, controlling for past 6-months nicotine and alcohol use, race, and age. Multiple comparisons were adjusted within models using the “sjstats” package for false-discovery rate corrections. Statistical decisions were made if corrected p (FDR-*p*) < 0.05.

### Post-hoc correlations

Given that recency and potency of cannabis use may influence cognitive performance, Pearson correlations were run between self-reported days since last episode of cannabis use and self-reported potency with each cognitive task. For those reporting on potency, when there was discrepant potency between flower and concentrate, the higher potency product was included; in addition, reports of “don’t know” for potency were excluded from analyses. As not all participants had used cannabis, the for rency of use was *n* = 184 and for potency was *n* = 150.

## Results

### Sociodemographics and substance use history

Full sociodemographic and substance use characteristics are displayed in Table 1. Mean age of participants was 19.5 years. Of the *n* = 79 participants who did not use cannabis regularly in the past 6-month, *n* = 41 had never used cannabis. Out of all participants, 48% had used cannabis flower that they believed was at least 15% THC in potency. To determine covariates, participants were divided into a regular cannabis using group and controls. Groups differed by sex (χ^2^=8.36, *p* = .004) and race (Fisher’s two-tailed *p* = .03), but not by any other sociodemographic characteristics.

**Table 1:** Sociodemographics and substance use characteristics by current, regular cannabis use status

	Current, Regular Cannabis Use (*n* = 129) M/% (SD) range	Controls (*n* = 96) M/% (SD) range
Age	19.69 (1.48) *16–22*	19.24 (1.68) *16–22*
Education	13.2 (1.36) *10–16*	12.92 (1.65) *9–16*
% Female	38%	58%
% Hispanic	40%	35%
% Caucasian	51%	49%
Days since last cannabis use	2.69 (4.49) *0–27*	103.5 (187.32) *1-1070* *n* = 55
Past month cannabis use (episodes)	45.67 (76.33) *0-798*	0.88 (2.17) *0–14*
Past 6-month cannabis use (episodes)	255.1 (372.77) *28-3557*	3.78 (5.98) *0–22*
Age of first regular cannabis use	17.74 (1.64) *13–22* *n* = 127	--
Past 6-month NTP use (episodes)	891.6 (2642.48) *0-23258*	497.1 (1462.26) *0-9835*
Past 6-month alcohol use (episodes)	26.88 (27.13) *0-145*	17.49 (22.76) *0–88*
% Using High Potency Flower (> 15% THC)	71%	17%
% Using High Potency Concentrate (> 60% THC)	66%	8%

Groups as presented here were not used in any primary or secondary analyses, but only for the selection of covariates

### Primary analyses

#### Emotional Stroop

**Fig. 1:**
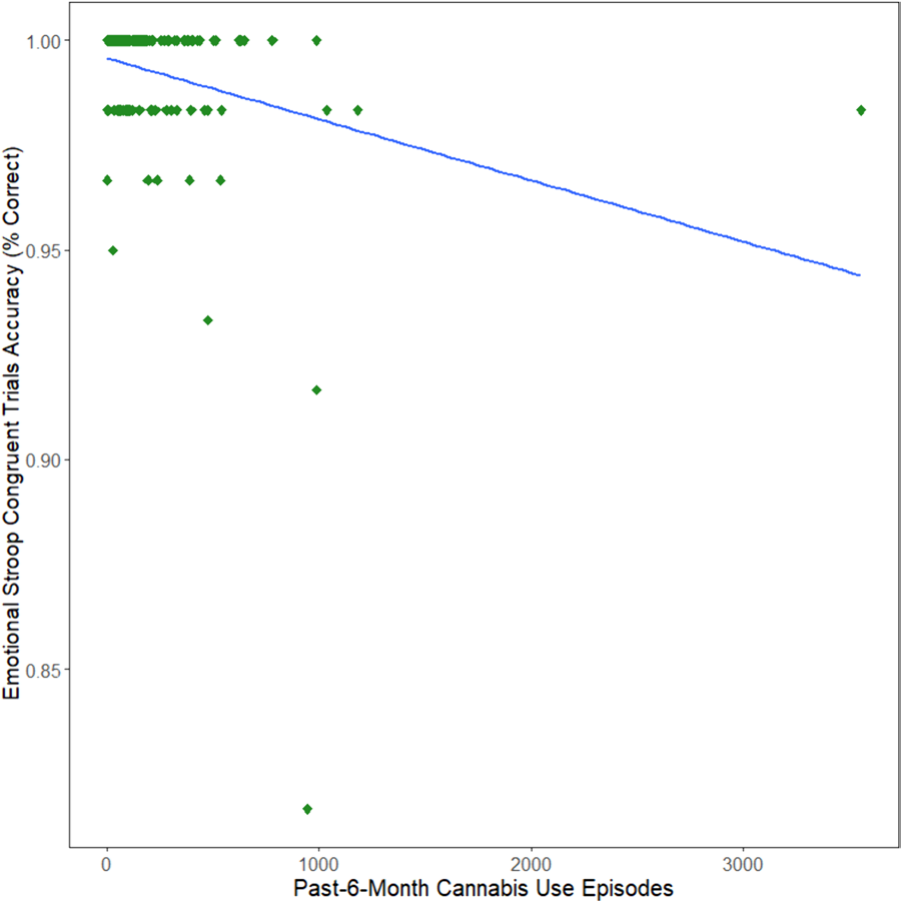
Scatterplot of Cannabis Use predicting Congruent Trial Accuracy. Greater past-6-month cannabis use negatively predicted decreased Emotional Stroop Congruent Trials Accuracy (percent of trials correct)

Regression analyses assessed past 6-month cannabis use episodes as a continuous variable in relation to four aspects of Emotional Stroop performance, controlling for nicotine use and alcohol use episodes, sex, and race; group status was not included in regressions. Past 6-month cannabis use was negatively associated with Congruent Trials Accuracy after correcting for multiple comparisons, such that greater cannabis use was associated with decreased accuracy (b = -0.00001, t = -3.56, *p* = .0004, FDR-*p* = .002; see Fig. 1). Congruent response time, incongruent accuracy, and incongruent response time were not significantly related to cannabis use.

#### Executive functioning

**Fig. 2:**
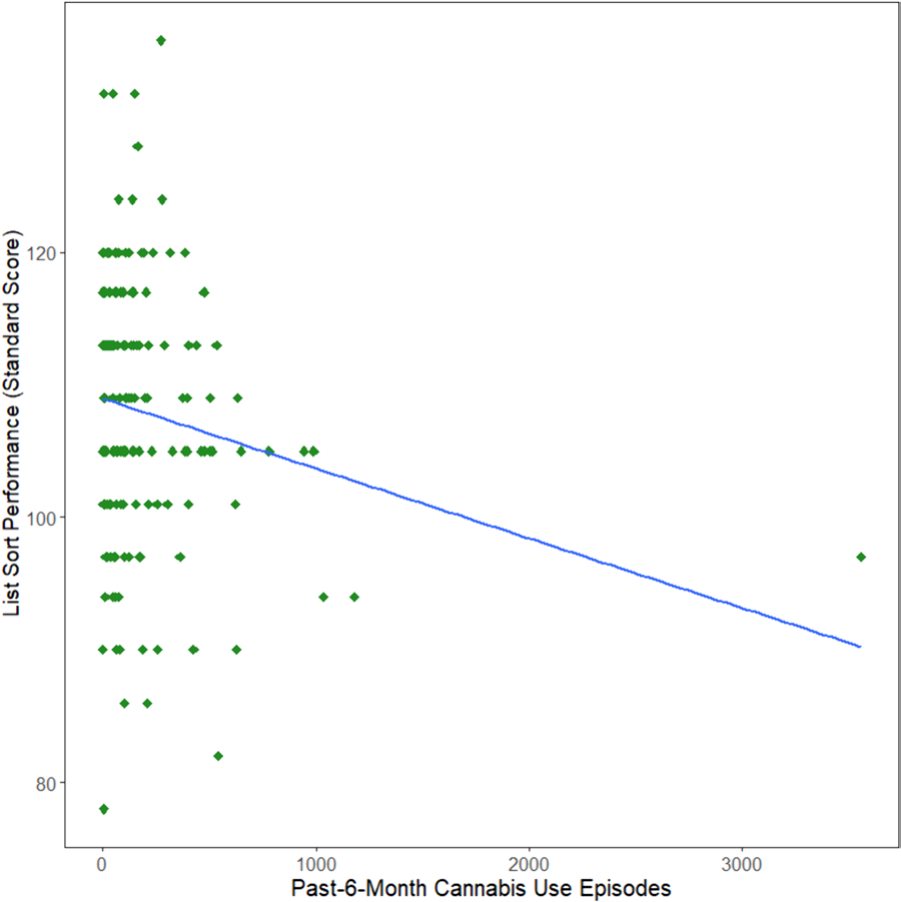
Scatterplot of Cannabis Use predicting List Sorting Performance. Greater past-6-month cannabis use marginally, negatively predicted decreased List Sort performance (standard score)

Greater past 6-month cannabis use was marginally associated with poorer List Sorting performance after correcting for multiple comparisons (b=-0.005, t=-2.33, *p* = .02, FDR-*p* = .10; see Fig. 2). There were no significant results for the models predicting Card Sorting, Picture Sequencing, or Pattern Comparison performance (*p’s* > 0.05).

### Secondary analyses

#### Age of onset of regular cannabis use

There were no significant associations between age of onset group and any of the nine scores for neurocognitive tasks. Prior to correcting for multiple comparisons, onset group significantly predicted Congruent Trials Accuracy performance (b = -0.004, t = -2.24, *p* = .03, FDR-*p* = .13), such that those who had regular onset of cannabis before age 18 demonstrated the poorest performance.

#### Sex at birth

No sex by past 6-month cannabis interactions were found to be significantly predictive of neurocognitive performance on any task.

### Post-hoc correlations

Correlations were run between days since last cannabis use (*n* = 184) and range of potency of THC (*n* = 150) with cognitive performance across tasks. No significant correlations were revealed.

## Discussion

The present study aimed to assess the relationship between cannabis use and emotional control and executive functioning in adolescents and young adults. Relationships emerged between increased past 6-month cannabis use and poorer accuracy on the Emotional Stroop and List Sorting tasks, though findings were modest after correction for multiple comparisons. Hypothesized relationships between age of onset of regular cannabis use with Emotional Stroop were similarly modest after corrections, and no relationships were significant with sex at birth and any task performance .

Here we used a newly validated Emotional Stroop paradigm [24] to test emotional functioning in emerging adults with cannabis use. This task jointly assesses behavioral inhibition, cognitive control, and emotional control. We found effects of past-6-month cannabis use relating to decreased congruent accuracy after correcting for multiple comparisons, indicating potential decrements in processing speed and attention during emotional processing associated with use. This is consistent with prior results in an overlapping yet different and smaller sample, where we similarly found decreased accuracy on the Stroop with increased cannabis use, though that study aimed to investigate the influence of NTP use [38]. Only one of four measures from the Emotional Stroop had demonstrated a significant relationship. This may suggest that emotional processing abilities, at least as assessed within this task, are less susceptible to cannabis use than anticipated from prior research on frontolimbic functioning in typically developing and medically healthy samples [1, 3, 13, 16, 17]. Notably, congruent accuracy performance is thought to measure processing speed, rather than pure emotional functioning [24]. As this task has only been recently validated as part of the Adolescent Brain Cognitive Development (ABCD) Study [24], it may also be that this task does not tap into socioaffective processing in young adults with cannabis use as sensitively as in largely substance naïve adolescents. This may also be supported by the modest relationships demonstrated between those younger age of regular use onset. However, the tasks may show more robust associations with substance use in those with heavier and more chronic use over the lifespan. Continued investigation into this and other tasks, particularly longitudinally, are needed to clarify cannabinoid-socioemotional functioning relationships.

List sorting performance was modestly associated with past-6-month cannabis use. The list sorting task was designed to tap into working memory abilities [39]—a construct frequently found to have cannabis-related deficits [40]. The endocannabinoid system is through to play an important role in modulating memory [41] due to the high density of CB1 receptors present in the prefrontal cortex, hippocampus, anterior cingulate, and cerebellum [42]. Thus, endogenous cannabis use likely impacts memory functioning and working memory abilities [43, 44]. Other executive functioning tasks were not associated with cannabis use; several reasons for this are considered. First, as summarized in a recent review [18], findings of modest decrements in select domains of cognitive functioning related to cannabis use in the developing brain are common. Importantly, though participants here included regular cannabis users, they may not be using typical products, as less than half of participants used cannabis with above average THC potency (15% THC as determined from seized samples, [45]). In addition, the present study used the NIH Toolbox, which is a validated research battery for neurocognitive assessment. Prior research has utilized clinical batteries (e.g., 14, 15), and other research in substance-using populations has suggested the NIH Toolbox may not be a sensitive battery for detecting substance and cognition relationships [46].

The secondary aim of the present analyses was to consider two commonly hypothesized factors of risk in brain-behavior relationships in cannabis use—age of onset of regular cannabis use and sex. However, despite predictions, there were no significant relationships between onset and sex subgroups and cognitive outcomes after corrections for multiple comparisons. For age of onset, this is in line with a systematic review that found only occasional age-dependent effects in both human and rodent samples [47]. For sex, it is intriguing that no sex differences in brain-behavior relationships emerged after corrections for multiple comparisons, despite a growing body of research indicating a range of sex-specific cognitive deficits in cannabis users [16, 22, 48, 49]. It may be that emerging adults with regular cannabis use are already outside the window of the highest vulnerability to the influence of cannabis rather than younger adolescents. Alternatively, while cannabis use was analyzed continuously, participants may be relatively light users early in their use trajectory (i.e., not using the majority of days in the past month) and therefore differences may still emerge with more chronic and longstanding use. Finally, other analyses from this sample have revealed largely intact white matter microstructure and cerebral blood flow [28, 29] as related to cannabis use, indicating that there may not be structural changes in these users that would warrant related functional changes in these youth still early in their use trajectories; however, this needs to be investigated directly.

Limitations to this study are noted. As described above, cannabinoid potency is rapidly changing [45] and may influence cognitive outcomes; however, here we relied on self-reported potency use and considered potency limitedly within this study. Participants may be using cannabis that is lower in THC potency than typically used in other samples. We also did not query cannabidiol (CBD) use, which may be another important factor in cannabinoid-brain-behavior relationships. While our investigation did not extend beyond the past 6 months of cannabis use, we acknowledge the significance of future research examining cannabis use trajectories, especially those individuals who exhibit patterns of heavy use followed by cessation [6]. Cannabis-use groups were used only to select covariates, with primary analyses conducted using episodic cannabis use information in linear regressions; secondary analyses used grouping variables (age of onset, sex) as interaction terms with continuous cannabis use information. We did not investigate cannabis use beyond past 6-months, though it is important for future research to consider cannabis use trajectories and, particularly, those who use heavily then cease use. As noted, neurocognitive measures were limited to more research-based assessments, which may not be as sensitive for changes in cognitive performance. Though MRI metrics were collected for many of these participants, they were not assessed here due to power concerns; future analyses will focus analysis on multimodal imaging metrics in conjunction with neurocognitive performance in this sample. Finally, findings may not generalize to other populations.

Taken together, we found modest evidence of associations between slower processing speed in an Emotional Stroop task (including in those with earlier age of regular use onset) and poorer working memory associated with more past 6-month cannabis use in adolescents and young adults. Yet, we found no other cannabis-cognition relationships, nor did we find other significant relationships by age of onset of regular cannabis use or sex at birth. Longitudinal studies which detail patterns of cannabis and other substance use are needed to better assess brain-behavior relationships and test the role that other factors (e.g., age of regular use, sex) play in these relationships.

## Data Availability

The datasets used and analysed during the current study are available from the corresponding author on reasonable request.
